# Betanodavirus B2 protein triggers apoptosis and necroptosis in lung cancer cells that suppresses autophagy

**DOI:** 10.18632/oncotarget.21588

**Published:** 2017-10-06

**Authors:** Hsuan-Wen Chiu, Yu-Chin Su, Jiann-Ruey Hong

**Affiliations:** ^1^ Department of Biotechnology and Bioindustry, Laboratory of Molecular Virology and Biotechnology, Institute of Biotechnology, National Cheng Kung University, Tainan 701, Taiwan; ^2^ Department of Biotechnology and Bioindustry, National Cheng Kung University, Tainan 701, Taiwan

**Keywords:** B2 protein, cell death, p53, autophagy, lung cancer cell

## Abstract

The betanodavirus B2 protein targets the mitochondria and acts as a “death factor”, but its effect on lung cancer cells is unknown. We examined the effect of the B2 protein on triggering apoptosis or necroptosis *via* P53-dependent and P53-independent pathways and increased in suppression of autophagy. The B2 protein targets the mitochondria of A549 (P53^+/+^) and H1299 (P53^—/—^) lung cancer cells due to a specific signal sequence (^41^RTFVISAHAA^50^). This triggers generation of reactive oxygen species within the mitochondria, and a minor stress response in A549 cells, but a strong stress response in H1299 cells. We examined the molecular mechanism of this cell death pathway, and found that B2 protein induces the P53/Bax-mediated apoptotic pathway in A549 cells, and that a P53 specific inhibitor (pifithrin-α) switches this response to RIP3-mediated necroptosis. On the other hand, B2 induces RIP3-mediated necroptosis pathway in H1299 cells, and a necroptosis inhibitor (necrostatin-1) switches this response to the apoptotic pathway. Both types of cell death signals inhibited autophagy *via* a tightly increased balance of beclin-1 and Bcl-2. Thus, B2 protein triggers P53-dependent apoptosis in A549 cells and ROS/RIP3-mediated necroptosis in H1299 cells, and crosstalk of these pathways limits initiation of autophagy. These findings provide new insights into the possible control and treatment of lung cancer.

## INTRODUCTION

Betanodaviruses are the causative agents of viral nervous necrosis (VNN) in fish, an infectious neuropathological condition characterized by necrosis of the central nervous system, including the brain and retina [1]. Clinical signs include abnormal swimming behavior and darkening of the fish [2]. VNN can cause massive dying off of the larval and juvenile populations of several marine teleost species [3]. Little is known about the molecular pathogenesis of VNN.

The nodavirus genome comprises two single-stranded molecules of positive polarity, RNA1 and RNA2 that are approximately 3.1 and 1.4 kb in length, respectively. RNA1 encodes a nonstructural protein of approximately 110 kDa, designated RNA-dependent RNA polymerase or protein A that is vital for replication of the viral genome. RNA2 encodes a 42-kDa capsid protein [4, 5] that may induce post-apoptotic necrotic cell death through a pathway mediated by cytochrome c release [6]. In RNA replication, betanodaviruses synthesize a sub-genomic RNA3 from the 3’ terminus of RNA1 that encodes two proteins, B1 and B2 [1, 7, 8]. In RGNNV, B1 plays anti-necrosis functions [9]. B2 acts as a host siRNA silencing suppressor in alpha- [10–12] and beta-nodavirus [7]. Recently, the B2 protein can induce oxidative stress-mediated cell death via mitochondrial targeting *in vitro* and *in vivo* [13].

The tumor suppressor protein P53 plays a major role in the cellular response to DNA damage and in protecting the genome from mutations. Activation of p53 can promote cell death or survival [14]. The P53 protein mediates cellular stress responses, in that it can initiate DNA repair, cell-cycle arrest, and senescence [15–18]. Importantly, P53 also regulates apoptosis, necroptosis, and autophagy [19]. When DNA repair fails, p53 initiates apoptosis by transactivating pro-/anti -apoptotic proteins that have roles in the signal transduction of apoptosis and necroptosis [20].

Apoptosis occurs normally during development and aging, and functions as a homeostatic mechanism to maintain cell populations in tissues. Apoptosis also functions as a defense mechanism, as in immune reactions or responses to cell damage from diseases or harmful agents. There are two major apoptotic pathways: the extrinsic (or death receptor) pathway and the intrinsic (or mitochondrial) pathway [21]. The extrinsic pathway is characterized by transmembrane receptor-mediated interactions, in which death receptors (members of the tumor necrosis factor [TNF] receptor gene superfamily) have a role [22]. The intrinsic pathway has a diverse array of non-receptor-mediated stimuli that produce intracellular signals, which act directly on targets within the cell, and are mitochondria-initiated events.

Recent studies indicate that necrosis is not just a series of unregulated processes, but is a series of programmed events, termed necroptosis [23]. In fact, TNFα, FasL, and TRAIL, the same ligands that activate apoptosis, can also stimulate necroptosis. Receptor interacting protein (RIP) kinases are also crucial regulators of cell survival and death [24]. There are seven proteins in the RIP family, each of which has a kinase domain (KD). Importantly, activation of RIP1 kinase regulates the necroptotic death pathway [25].

Autophagy is a highly conserved catabolic process, in which there is degradation of proteins and organelles that promote survival or death, depending on the physiological and pathological conditions [26]. A key part of autophagy is the sequestration of proteins and organelles within double-membrane structures, termed autophagosomes. Lysosomes target the autophagosomes, which degrade them to autophagic vacuoles or autophagolysosomes. Induction of several autophagy-related genes, including LC3, phosphatidylinositide 3-kinase, and Beclin 1 (which is regulated by Bcl-2 and Bcl-xL proteins) [27–29], initiates the formation of an autophagosome.

We previously studied the effect of B2 protein on ATP depletion-induced cell death *in vitro* and *in vivo* [13, 30, 31] in a line of fish cells and a zebra fish model system. However, the effect of the B2 protein on the cell death pathways in lung cancer cells is still unclear. In this study, we used the novel viral B2 protein to induce different cell death pathways in A549 lung cancer cells, which express P53 (P53^+/+^), and H1299 lung cancer cells, which do not express P53 (P53^—/—^), and also examined relationship of activation of these different pathways with suppression of autophagy. These data may provide new insight into the control and treatment of lung cancer.

## RESULTS

### B2 protein targets lung cancer cell mitochondria

**Figure 1:**
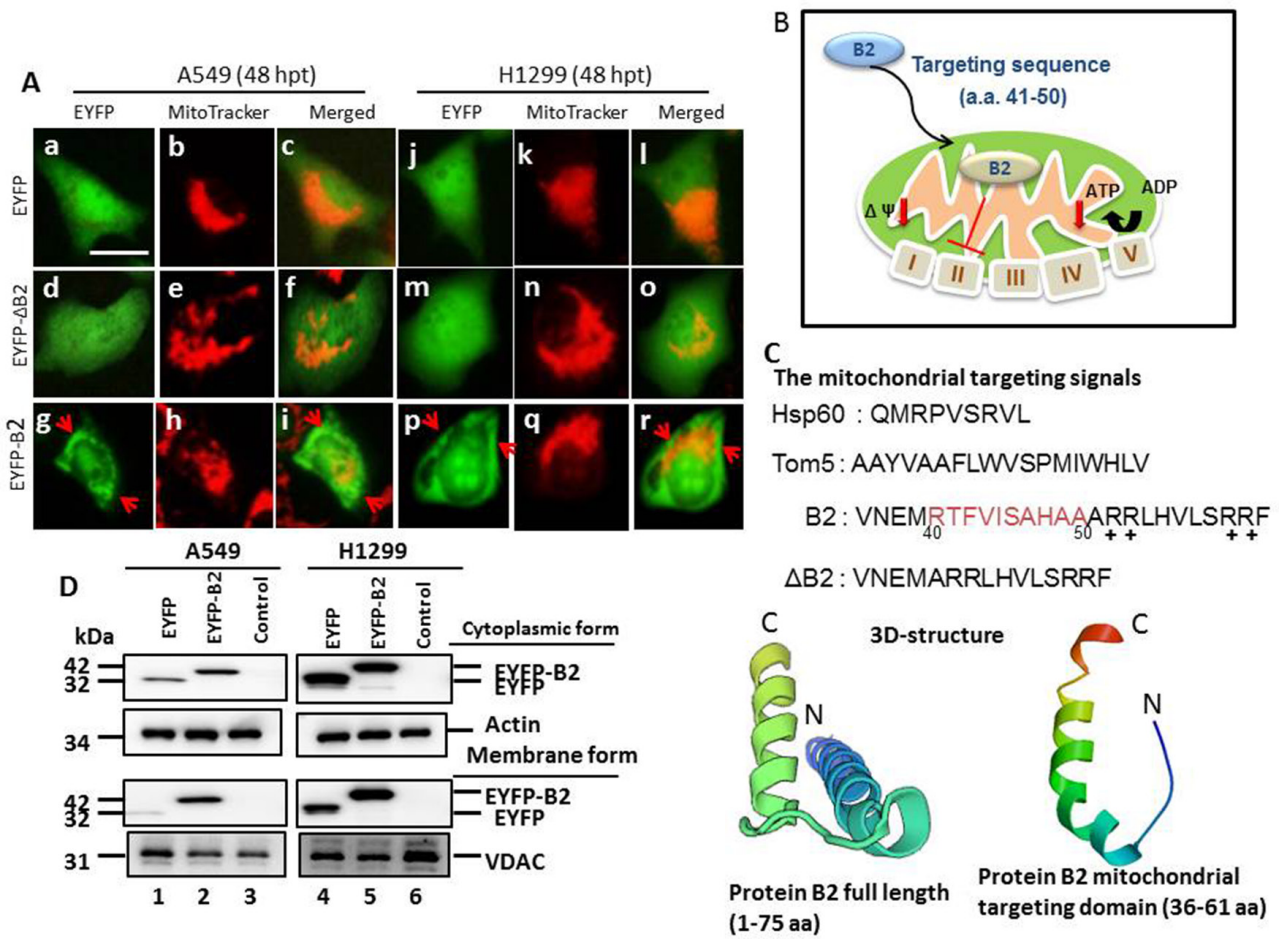
MitoTracker staining indicates the RGNNV B2 protein targets the mitochondria in human lung cancer cells Analysis of mitochondrial targeting of the EYFP-B2 fusion protein at 48 h post-transfection indicated yellow fluorescence in ∼4-5% of A549 cells (**A**: g-i and d; indicated by arrows) and H1299 cells (A: p-r) relative to cells with EYFP (A: a-c, in A549 cells; A: j-l, in H1299 cells) and EYFP-ΔB2 (del^41^RTFVISAHAA^50^) (A: d-f, in A549 cells; A: m-o, in H1299 cells). Phase-contrast images of EYFP-B2 transfected cells at 36 h post-transfection shows that the EYFP-B2 fusion protein targets mitochondria (indicated by arrows; A: i in A549 cells; A: r in H1299 cells). Scale bar: 10 μm. **(B)** RGNNV B2 protein construct used for mitochondrial targeting. **(C)** Various constructs of wild type and mutant forms of the RGNNV B2 protein used to identify the mitochondrial targeting sequence. The 3D-structure of full length of RGNNV protein B2 (1-75 aa) and B2 mitochondria targeting domain (36-61 aa) alone (see Materials and Methods) were shown, and that alpha helix also existing. N: N terminus; C: C terminus. **(D)** Immunoblotting using monoclonal antibodies against EYFP shows the protein distribution in mitochondrial and cytosolic fractions at 48 h post-transfection. The internal controls were actin (cytosolic fraction) and VDAC (mitochondrial membrane fraction). EYFP alone (negative control; lanes 1 and 4); EYFP-B2 (lanes 2 and 5), controls without vector (A549 and H1299 cells; lanes 3 and 6).

The betanodavirus B2 protein targets mitochondria *via* a specific signal peptide (^41^RTFVISAHAA^50^) [13]. We determined if the B2 protein can also target the mitochondria of human lung cancer cell lines A549 (P53^+/+^) and H1299 (P53^—/—^). Thus, we used full-length EYFP-B2 and EYFP-ΔB2, which has a deleted targeting region (Figure 1B and 1C) that further have predicted the 3D-structure of full length (1—75 aa) and B2 mitochondria targeting domain (36 aa) as a major alpha helix confirmation, and measured localization of B2 protein using MitoTracker and measurement of green fluorescence. The results show green fluorescence in the mitochondria of cells transfected with the full-length EYFP-B2 (Figure 1A and 1Ag-1Ai: A549 cells; p-r: H1299 cells). In contrast, cells of the EYFP group (Figure 1A and Figure 1Aa-1Ac: A549 cells; d-f: H1299 cells) and the EYFP-ΔB2 group (Figure 1A and Figure 1Aj-1Al: A549 cells; m-o: H1299 cells) have green fluorescence almost entirely in the cytoplasm. We also examined the mitochondrial localization of B2 by performing western blotting analysis at 48 h post-transfection (Figure 1D). These results confirm that B2 targets the mitochondria in A549 and H1299 cells that were transfected with the full-length EYFP-B2, but not in cells of the other groups.

### B2 protein induces stronger ROS production in H1299 (P53^—/—^) cells than A549 (P53^+/+^) cells

**Figure 2:**
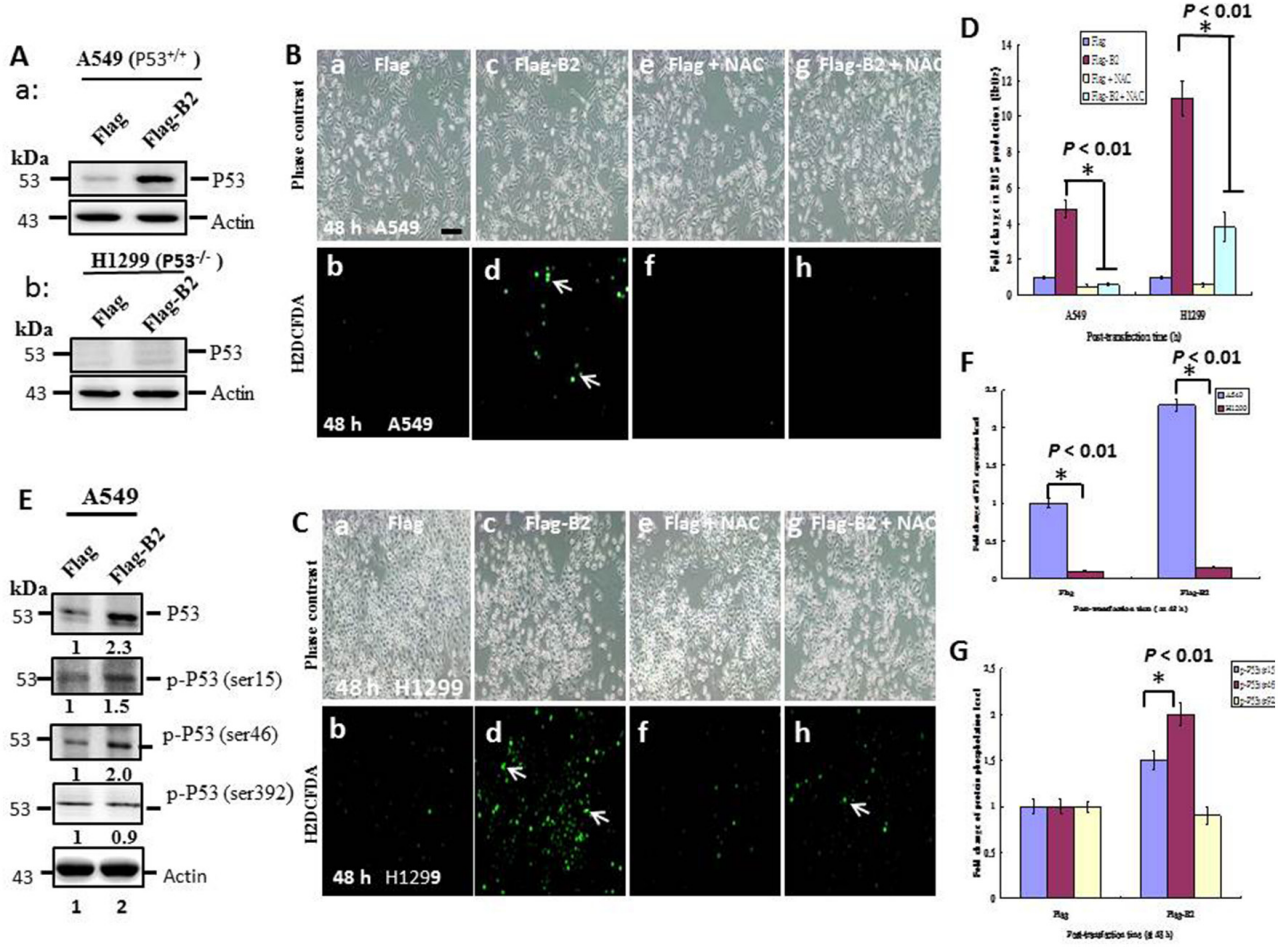
Targeting of B2 protein to mitochondria induces stronger ROS production in H1299 cells than A549 cells **(A)** Immunoblotting shows the expression of P53 in A549 cells (P53^+/+^) and H1299 cells (P53^—/—^) at 48 h post-transfection, with actin used as an internal control. ROS production (arrows) by A549 cells **(B)** and H1299 cells **(C)** at 48 h post-transfection. In B and C, FLAG, negative control (a and b); FLAG-B2 (c and d); FLAG + N-acetylcysteine (NAC) (e and f), FLAG-B2 + NAC (g and h). Green fluorescence indicates ROS production. Scale bar = 20 μm. **(D)** Quantification of the data in B and C. Error bars represent the SEM of 3 independent experiments. All data were analyzed using a paired or unpaired Student’s *t*-test, as appropriate. ^*^p < 0.01 indicates statistical significance. **(E)** Identification of P53 expression and phosphorylation sites in A549 cells by western blot analysis. **(F** and **G)** Quantification of the results in A and E, respectively. Error bars represent the SEM of 3 independent experiments. All data were analyzed using a paired or unpaired Student’s *t*-test, as appropriate. ^*^p < 0.01 indicates statistical significance.

Previous studies of fish indicated that B2 protein targeting of mitochondria correlates with ROS production [30]. Thus, we measured B2-induced generation of ROS at 48 h post-transfection in both lines of cancer cells. We also determined P53 expression in A549 cells (Figure 2A and 2Aa: lane 1) and H1299 cells (Figure 2A and 2Ab: lane 1) at 48 h post-transfection. The results show that FLAG-B2 expression had a stronger increasing on ROS production in H1299 cells (Figure 2C, 2Cc and 2Cd) than in A549 cells (Figure 2B, 2Bc and 2Bd) based on the H2DCFDA assay that inhibited by antioxidant inhibitor NAC (Figure 2C, 2Cg and 2Ch in H1299 cells; Figure 2B, 2Bg and 2Bh in A549 cells). Relative to cells treated with NAC, there was a 4-fold increase of ROS generation in A549 cells, and a 6-fold increase in H1299 cells (Figure 2D). We also found that B2- induced ROS production upregulates P53 by ∼1.3-fold in A549 cells (Figure 2E, lane 2 and 2F). Moreover, B2-induced ROS significantly increased P53 phosphorylation on serine residue 15 (for DNA damage) and serine residue 46 (for apoptosis), but not serine residue 392 (for tumor induction) [16, 17].

### B2-induces apoptosis in A549 (P53^+/+^) cells and necroptosis in H1299 (P53^—/—^) cells

**Figure 3:**
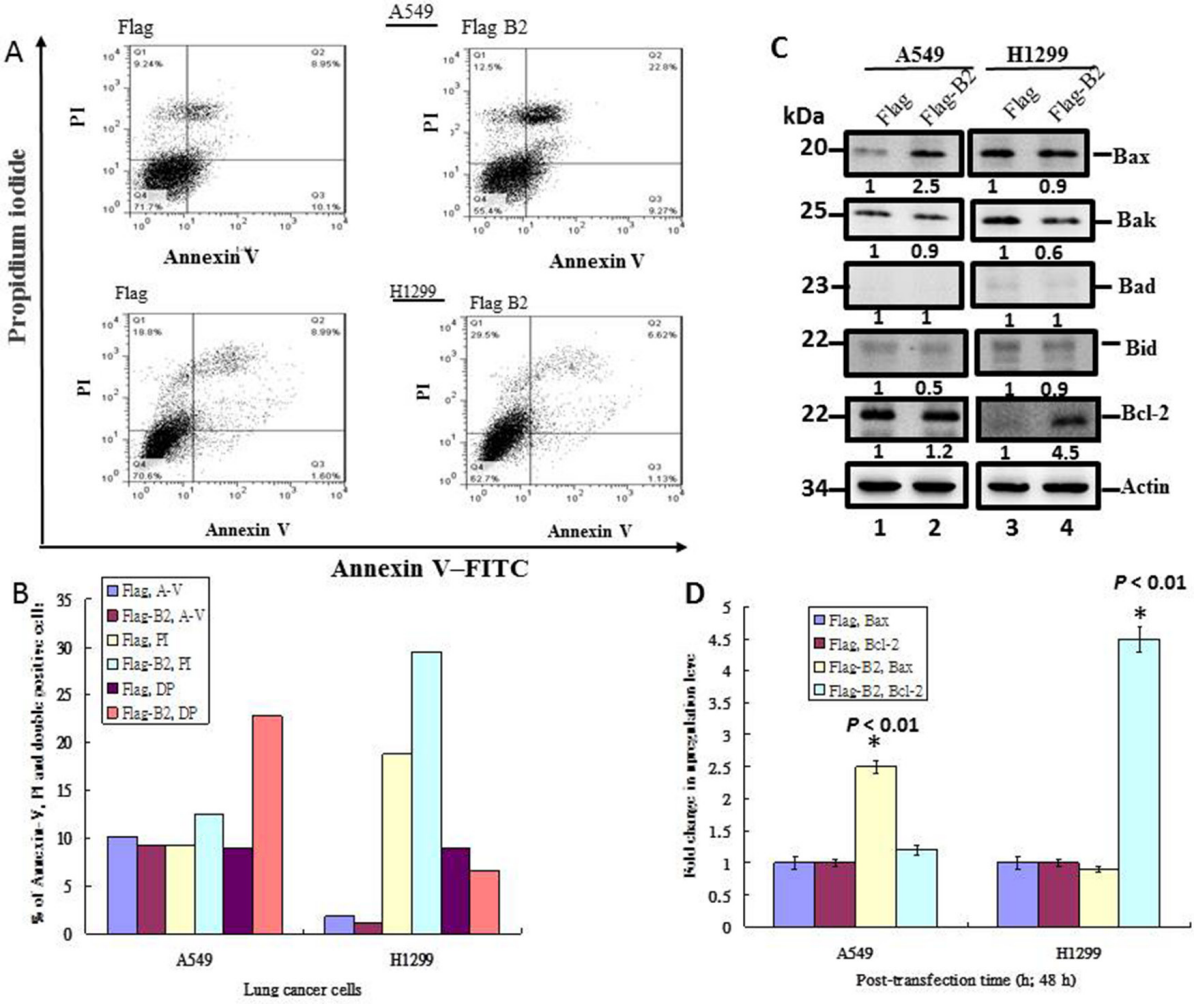
B2 protein induces Bax-mediated apoptosis in A549 cells, but induces RIP3-mediated necroptosis in H1299 cells **(A)** Representative flow cytometry results at 48 h post-transfection. Fluorescence of Annexin-V and PI were measured in 10,000 cells. Annexin-V-FITC^+^ cells indicate early apoptosis and PI^+^ cells indicate late apoptotic/secondary necrosis. **(B)** Quantitation of the percentage of viable cells (Annexin-V-FITC^+^ and PI^+^) from flow cytometry experiments. **(C)** Immunoblot analysis of A549 and H1299 cells using monoclonal antibodies against pro-apoptotic and anti-apoptotic proteins shows the expression of various forms of Bax, Bak, Bad, Bid and Bcl-2. ß-actin was a loading control. **(D)** Quantitative analysis of the pro-apoptotic and anti-apoptotic proteins from Figure 3C. Error bars represent the SEM of 3 independent experiments. All data were analyzed using a paired or unpaired Student’s *t*-test, as appropriate. ^*^P < 0.01 significantly different from the control.

Next, we determined the mechanism(s) by which the B2 protein induces cell death in both lines of lung cancer cells by use of flow cytometric analysis with Annexin-V-FITV and PI staining (Figure 3). The results show that B2 protein induces apoptosis in 13% of A549 cells (Figure 3A and 3B), but induces necroptosis in 10% of H1299 cells (Figure 3A and 3B).

We also found that B2 increased the expression of the pro-apoptotic gene Bax by ∼2.5-fold (Figure 3C, lane 2) in A549 cells, but not in H1299 cells (Figure 3C, lane 4). On the other hand, B2 increased the expression of Bcl-2 in H1299 cells by ∼4.5-fold, but only by ∼20% in A1299 cells (Figure 3D). In summary, B2 protein induces Bax-mediated apoptosis in A549 cells, but induces RIP3-mediated necroptosis in H1299 cells.

### A specific P53 inhibitor (pifithrin-α) leads to B2 protein-induction of necroptosis in A549 cells

**Figure 4:**
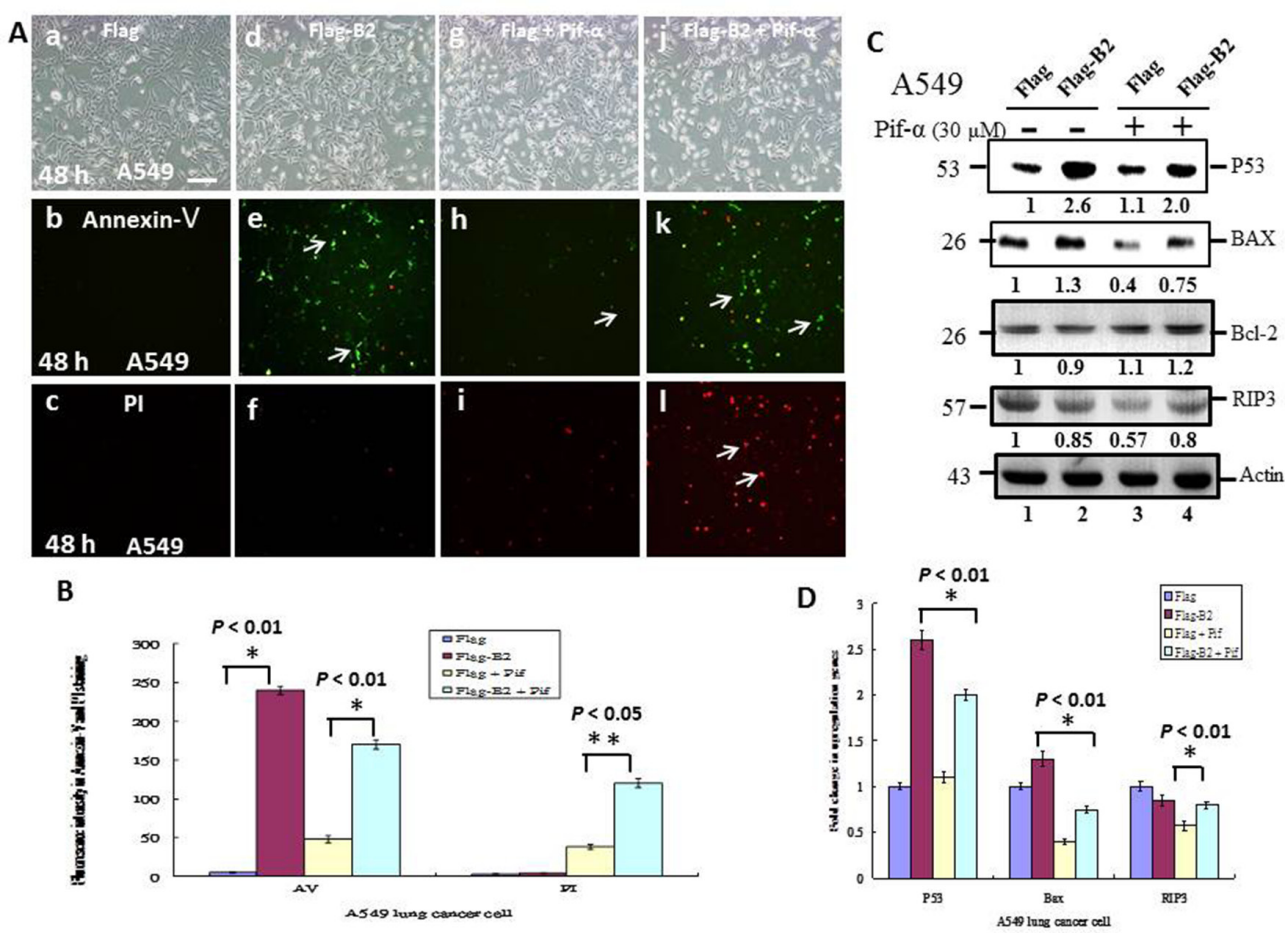
A P53 inhibitor blocks apoptosis and promotes necroptosis in A549 cells **(A)** Phase-contrast-fluoresence microscopy of cells in the FLAG group (a-c), FLAG-B2 group (d-f), FLAG + Pif α group (g-i) and FLAG-B2 + Pif α (30 μM) group (j-l) stained with Annexin-V-FITF (b, e, h, and k) and PI (c, f, i, and l) at 48 h post-transformation. **(B)** Quantification of fluorescence of Annexin-V and PI. Error bars represent the SEM of 3 independent experiments. All data were analyzed using a paired or unpaired Student’s *t*-test, as appropriate. ^*^P < 0.05 indicates statistical significance. **(C)** Influence of a P53 inhibitor on expression of apopotic and necrotic genes in A549 cells at 48 h post-transfection (western blotting). **(D)** Quantitation of western blotting results. Error bars represent the SEM of 3 independent experiments. All data were analyzed using a paired or unpaired Student’s *t*-test, as appropriate. ^*^P < 0.01 indicates statistical significance.

Next, we determined the role of P53 on apoptosis and necroptosis by double staining A549 cells with PI and annexin V-FITC, and treatment with a specific inhibitor of P53 (Pifithrin-α, 30 μM) (Figure 4). The results show that Pifithrin-α blocked P53 activity (Figure 4A and 4Ag-4Ai) and switched the cells from apoptosis (Figure 4A and 4Ad-4Af) to necroptosis (Figure 4A and 4Aj-4Al). Quantitative analysis of fluorescence intensity (Figure 4B) confirmed these differences were significant (*p* < 0.05 for all comparisons).

We also analyzed markers of apoptosis and necroptosis in A549 cells (Figure 4C and 4D). The results show that B2 induces a ∼1.3-fold increase in the pro-apoptotic gene Bax (Figure 4C, lane 2) and that Pifithrin-α treatment blocked this effect. Blockage of P53 activity was also associated with a ∼23% increase in RIP3 expression (Figure 4C, lane 4 and 4D). These differences were statistically significant (*p* < 0.01 for all comparisons).

### A specific inhibitor of necroptosis (necrostatin-1) leads to B2 protein-induction of apoptosis in H1299 cells

**Figure 5:**
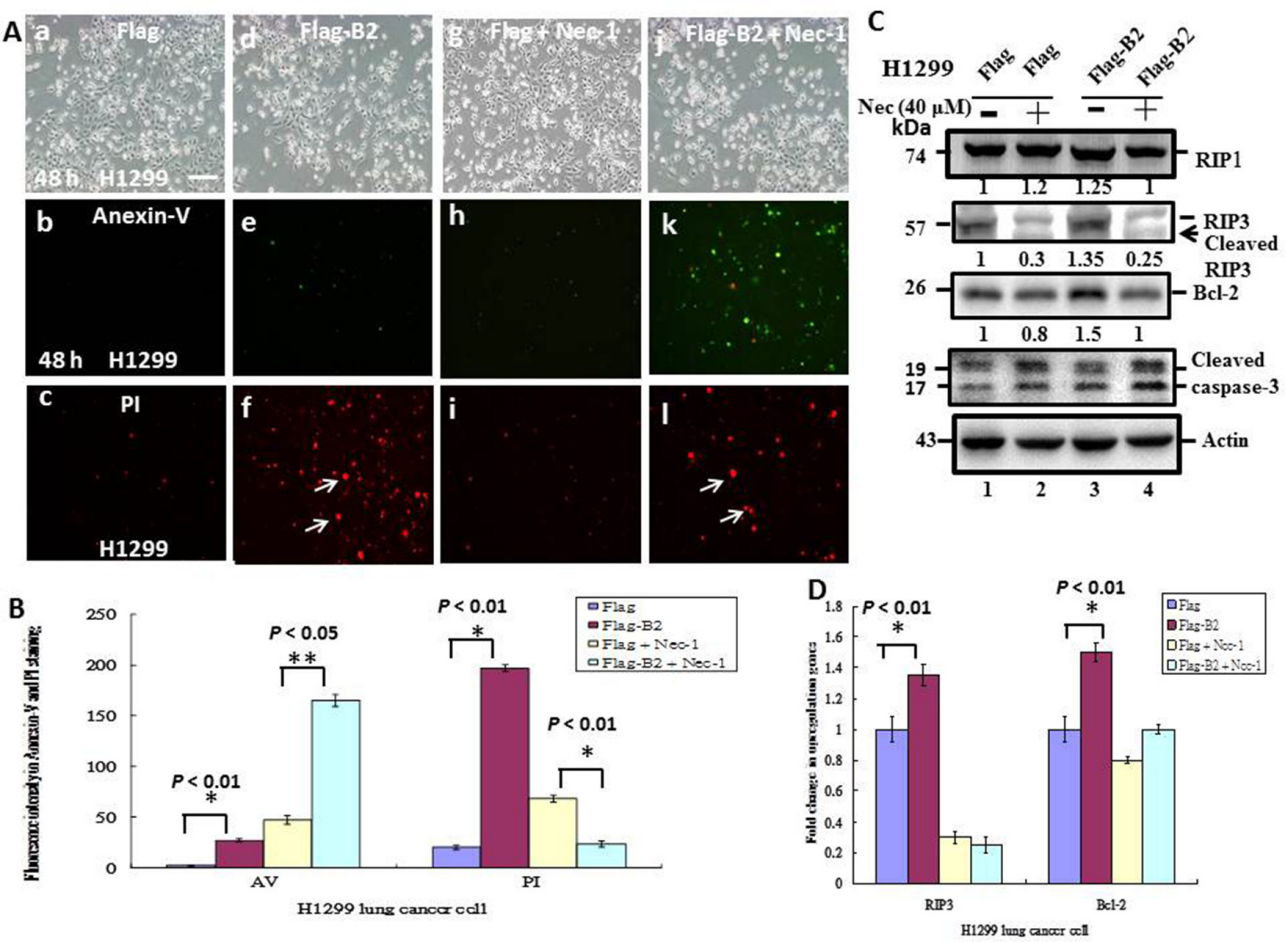
Blockage of RIP3 function switches B protein-transfected H1299 cells from necroptosis to apoptosis **(A)** Representative phase-contrast-fluoresence images of the FLAG group (a-c), FLAG-B2 group (d-f), FLAG + Nec-1 group (g-i) and the FLAG-B2 + Nec-1 group (j-l), with staining by Annexin-V-FITC (b, e, h, and k) and PI (c, f, i, and l) at 48 h post-transfection. **(B)** Quantification of FITC and PI fluorescence. Error bars represent the SEM of 3 independent experiments. All data were analyzed using a paired or unpaired Student’s *t*-test, as appropriate. ^*^p < 0.05 indicates statistical significance. **(C)** Influence of a necrosis inhibitor on expression of apoposis- and necrosis-related genes, based on western blot analysis at 48 h post-transfection. **(D)** Quantification of the western blotting results from C. Error bars represent the SEM of 3 independent experiments. All data were analyzed using a paired or unpaired Student’s *t*-test, as appropriate. ^*^p < 0.01 indicates statistical significance.

We also used a specific inhibitor of necroptosis (necrostatin-1, 40 μM) to determine the effect of blocking necroptosis in H1299 cells (Figure 5). Necrostatin-1 inhibits RIPK1 by blocking its association with RIP3. Based on the PI/Annexin V double staining assay, we found that treatment of H1299 cells with necrostatin-1 inhibited necroptosis, and increased apoptosis (Figure 5A, 5Ad-5Af and 5Aj-5Al). Quantification of green fluorescence (Annexin-V-FITC) with red fluorescence (PI) confirmed that this difference was significant (*p* < 0.01).

As previously, we also examined markers of apoptosis and necroptosis in H1299 cells (Figure 5C and 5D). The results show that B2 protein upregulated the necrosis gene RIP3 by ∼1.35-fold and Bcl-2 by ∼1.5-fold (Figure 5C, lane 3), and that necrostatin-1 blocked this upregulation (Figure 5C, lane 4). Necrostatin-1 blocks the association of RIP1 with RIP3, leading to RIP3 instability, and cleavage from a 57-kDa to a 55-kDa protein (Figure 5C, lane 2 *vs.* lane 4). On the other hand, necrostatin-1 blockage of necroptosis increased the number of H1299 cells undergoing apoptosis. Quantitation of these results indicates the differences were significant (*p* < 0.01 for all comparisons).

### B2 protein regulates cell death, but limits induction of autophagy by maintaining a balance of beclin-1 and Bcl-2 in lung cancer cells

The interaction of B2 protein-induced cell death with autophagy is unknown. We examined the effect of B2 protein-induced cell death on autophagy by treatment of cells with the P53 inhibitor Pifithrin-α or the necrotsis inhibitor necrostatin-1.

**Figure 6:**
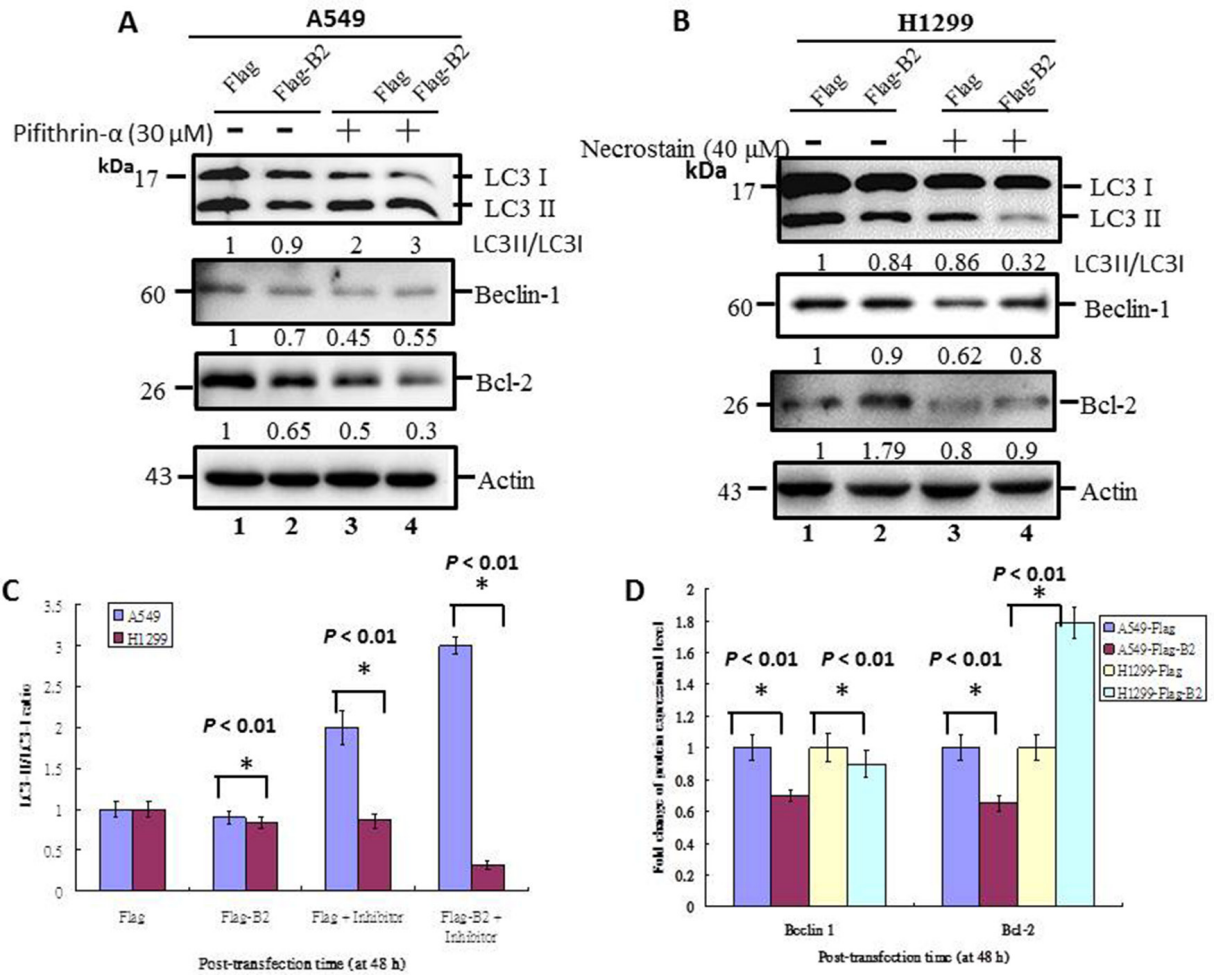
Crosstalk of apoptosis and necroptosis pathways limits initiation of autophagy in human lung cancer cells Immunoblotting at 48 h post-transfection using monoclonal antibodies against autophagy-related proteins (LC3-I, LC3-II, Beclin-1, and Bcl-2) in A549 cells, with and without a P53 inhibitor **(A)** and in H1299 cells, with and without a necrosis inhibitor **(B)**. ß-actin was used as a loading control. **(C** and **D)** Quantification of the results in A and B, respectively. Error bars represent the SEM of 3 independent experiments. All data were analyzed using a paired or unpaired Student’s *t*-test, as appropriate. ^*^p < 0.01 indicates statistical significance.

Thus, at 48 h post-transfection, B2 expression inhibited autophagy initiation in A549 cells (Figure 6A) and H1299 cells (Figure 6B) based on the LC3-II/LC3-I ratio, a marker of autophagy. There was also downregulation of the autophagy regulation genes, beclin 1 and Bcl-2, in A549 cells, although there was upregulation of Bcl-2 and downregulation of beclin-1 in H1299 cells. Blockage of P53 in A549 cells increased the LC3-II/LC3-I ratio (Figure 6A, lane 4), and this correlated with increased expression of beclin1 and decreased expression of Bcl-2. In contrast, blockage of necrosis in H1299 cells is inhibted autophagy (Figure 6B, lane 4) on LC3-II/LC3-I ratio (Figure 6B, lane 4), and increased minor upregulation of Bcl-2 and beclin 1. Quantification of these results indicated the differences were significant (*p* < 0.01 for all comparisons) (Figure 6C and 6D). In summary, these experiments indicate the P53 gene has a role in supression of autophagy in A549 cells, but not in H1299 cells.

## DISCUSSION

B2 protein expression induces necroptosis and breakdown of mitochondria in aquatic fish cells, and this correlates with B2 targeting of mitochondria, ROS generation, and ATP depletion from complex-V (F_0_F_1_-ATP synthase). Furthermore, B2 protein induces cell death in zebrafish during the early embryonic stage (within 12 h post-infection) [13, 30, 31]. These results suggest that transfection experiments in which B2 protein expression is induced in other types of cells, such as lung cancer cells, may help to elucidate the mechanisms of cell death.

The present study examined the effect of the non-structural protein B2 as a novel “death factor” that targets mitochondria and regulates apoptosis and necroptosis in lung cancer cells, depending on the presence of P53. We found that B2 protein targets the mitochondria of lung cancer cells, and that targeting increases to ROS production and apoptosis in A549 cells (P53^+/+^) and necroptosis in H1299 cells (P53^—/—^). Our results also suggest that the presence of pathways for two types of cell death – apoptosis and necroptosis -- limits the extent of autophagy in these cells.

### B2 protein targets mitochondria and triggers stress signals in lung cancer cells

Our results indicate that the B2 signal region was between amino acids 41-50 (^41^RTFVISAHAA^50^), and includes 10 amino acids [13] that target the mitochondria of A549 and H1299 cells (Figure 1B). This signal region is different from the signal regions of other proteins that target mitochondria (HSP60 and TOM5; Figure 1C) that protein B2 targeting domain was shown the alpha helix structure. Our results also show that removal of this signal peptide blocked the ability of B2 protein to target mitochondria (Figure 1A). Furthermore, we found that B2 mitochondrial targeting increases to ROS production in both cell types, although with a there was a 4-fold increase of ROS in A549 cells and an 8-fold increase of ROS in H1299 cells (Figure 2). Thus, P53 appears to have a more important role in directly regulation of oxidative stress A549 cells, but additional factors may also regulate stress in H1299 cells.

### B2 protein induces ROS-mediated stress signals that regulate P53 expression and phosphorylation

P53 is a master controller of cellular responses, and functions as a ‘guardian of the genome’ [32]. Most cancers show loss of p53 function [33–37], underscoring its importance in tumor suppression. P53 alters the expression of many genes involved in specific cellular responses, including cell cycle arrest, senescence, and apoptosis [32, 36, 38–42], although there is an incomplete understanding of the factors that determine cell fate after p53 upregulation [43]. We found that B2-triggered ROS production correlates with upregulation of P53 and the downstream gene Bax (Figure 3C) in A549 cells (P53^+/+^), but not in H1299 cells (P53^—/—^) (data not shown). B2-triggered ROS signals increase P53 protein phosphorylation at Ser15 (for DNA damage) and Ser46 (for apoptosis), but not at Ser93 (for proliferation) (Figure 2E-2F).

Furthermore, we found that B2 protein expression has different effects on cells with different genetic backgrounds (*i.e.* A549 cells (P53^+/+^) and H1299 cells [P53^—/—^]). B2 protein triggers apoptosis through P53/Bax signaling in A549 cells (Figure 4) [30, 31]. However, blockage of P53 function in A549 cells by a specific inhibitor switched the mechanism to necroptosis, as indicated by downregulation of Bax and upregulation of RIP3 (Figure 4C). By contrast, we found that B2 protein triggers necroptosis *via* the ROS/RIP3- pathway in H1299 cells (Figure 5), and that treatment of these cells with a specific inhibitor of necroptosis switched to mechanism to apoptosis (Figure 5C), as indicated by downregulation of Bcl-2 expression.

### Induction of two cell death pathways by B2 protein inhibits initiation of autophagy

In normal physiological situations, autophagy is always occurring at a basal level, and it functions as an intracellular quality-control system that maintains homeostasis by removal of superfluous and/or damaged proteins [34–46]. Autophagy and apoptosis both occur when cells are under stress [46]. Normally, autophagy precedes apoptosis and maintains cell homeostasis, with a tight crosstalk between these pathways. Some factors function in apoptosis and autophagy, such as Beclin-1 and Bcl-2, that protein interaction on control to apoptosis or autophagy was required [47].

We found that B2 triggered P53/Bax-mediated cell death *via* apoptosis in A549 lung cancer cells, and that crosstalk with the autophagy pathway occurs through downregulation of Beclin-1 and Bcl-2 (Figure 6A) [46, 48]. Furthermore, inhibition of P53 by Pif-α switched the cells from apoptosis to autophagy *via* minor Beclin-1 upregulation and minor Bcl-2 downregulation. This indicates that P53-mediated apoptosis can directly regulate autophagy. On the other hand, B2 induces ROS/RIP3-mediated cell death *via* necroptosis in H1299 cells, and also limits autophagy *via* minor Beclin-1 downregulation and strong Bcl-2 upregulation (Figure 6B). Then, further necroptotic process inhibited by necroptosis inhibitor Nec-1 was found that minor double upregulated the Beclin-1 and Bcl-2 expression, which necroptotic death signals is involved in autophagy regulation.

**Figure 7:**
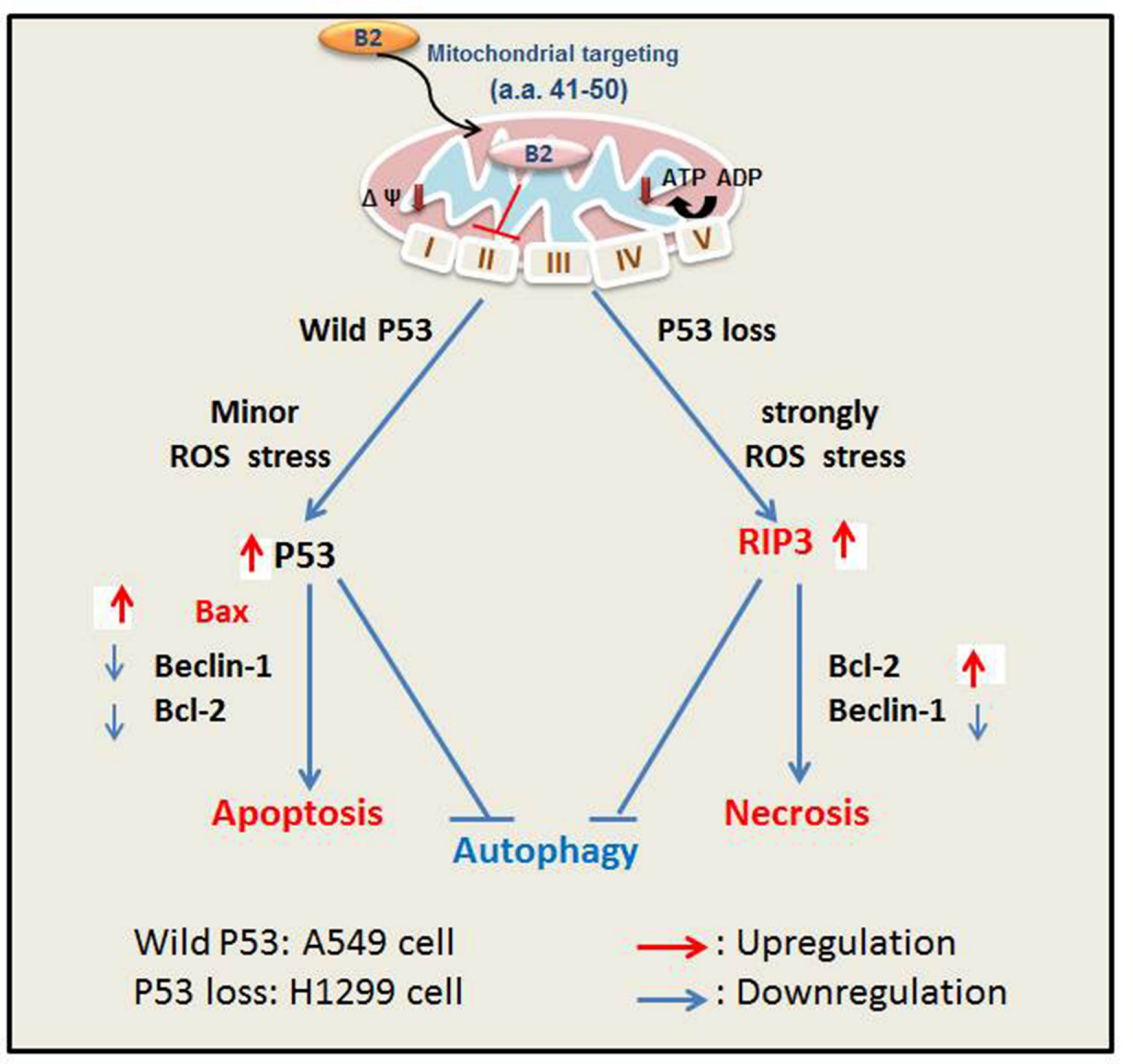
Hypothesized effect of protein B2-induced ROS-mediated apoptosis (A549 cells, P53+/+) and necroptosis (H1299 cells, P53—/—) Transfection of cells with RGNNV B2 protein induces ROS-mediated stress, leading to apoptosis (A549 cells) or necroptosis (H1299 cells). B2 protein first localizes to the mitochondria due to its targeting signal ^41^RTFVISAHAA^50^ (3). B2 protein then modulates complex II activity, reducing ATP levels, and increasing ROS production. Minor ROS stress upregulates P53 and leads to apoptosis in A549 cells; strong ROS stress increases ROS/RIP3-mediated necrotic cell death in H1299 cells. Crosstalk between these two signaling pathways prevents autophagy, due to a balance of beclin-1 and Bcl-2. The P53/Bax-mediated cell death signaling limits autophagy due to downregulation of Beclin 1 and Bcl-2 in A549cells, but treated with P53 inhibitor pifithrin-α can enhance autophagy; ROS/RIP3 death signaling limits autophagy *via* upregulation of Bcl-2 and downregulation of Beclin-1 in H1299 cells, but treated with a specific inhibitor of necroptosis Nec-1 can downregulate Bcl-2 for more reducing initiation of autophagy.

In summary, we found that the B2 protein triggers death of lung cancer cells *via* promotion of P53/Bax-mediated apoptosis (A549 cells) and by ROS/RIP3-mediated necroptosis (H1299 cells) (Figure 7). Moreover, ROS generation and metabolism has roles in the P53-dependent and P53-independent pathways [49]. Our findings also suggest that crosstalk of the apoptosis and necroptosis pathways can reduce autophagy by altering the balance of beclin-1 and Bcl-2. If P53 activity is blocked, then Rif-α can switch from apoptosis to necroptosis, with only minor promotion of autophagy in A549 and H1299 cells. On the other hand, we found that blockage of necroptosis switches cells to apoptosis, but with no apparent initiation of autophagy in H1299 lung cancer cells, in contrast to A549 cells. Our findings indicate that B2 protein induces two cell death pathways – a P53-dependent pathway and a P53-independent pathway. Thus, the genetic background of a cell determines which pathway is triggered, a death signals to regulate autophagy initiation between beclin-1/Bcl-2 interaction, and this provides new insight into cancer control and therapy.

## MATERIALS AND METHODS

### Cell culture

Two human non-small cell lung cancer cell lines were used for experiments: the epithelial cell line A549 (ATCC, CCL-185™; with wild type P53 expression [P53^+/+^]) and line NCI-H1299 (ATCC, CRL-5803™; without P53 expression [P53^—/—^]). Cells were cultured in DMEM medium supplemented with 10% fetal bovine serum, 100 units/mL penicillin, and 100 μg/mL streptomycin (Invitrogen) at 37°C in an atmosphere of 5% CO_2_ in 10 cm^2^ Petri dishes or 6 well culture plates.

### Plasmid construction

The RGNNV B2 gene with targeting sequence (^41^RTFVISAHAA^50^) or deleted fragments were cloned into the pcDNA3.1 vector (Clontech Laboratories, Palo Alto, CA), the p3XFLAG-*myc*-CMV-26 vector (Sigma), and the pEYFP-C1 vector (Clontech), with the enhanced yellow fluorescent protein (EYFP) [13].

### Cell transfection

Polyethylenimine (PEI; Sigma Aldrich, 408727) was used as the transfection agent [50, 51]. For cell transfection, 4×10^5^ cells were seeded in 6-well culture plates. On the following day, 3.2 μg of recombinant plasmid was mixed with 3.2 μg of PEI, and the transfection procedure was carried out according to the manufacturer’s instructions.

### Preparation of mitochondria from B2-transfected cells

A549 and H1299 cells were seeded in 60-mm diameter culture dishes with 4 mL of medium (10^5^ cells/mL) for 24 h. These cells were then transfected with EYFP or EYFP-B2 for 48 h. At each change of the culture medium, 1 mL of medium was removed. Mitochondria were isolated by modification of a previously described protocol [30]. Briefly, cells (2×10^6^) were washed with PBS and homogenized in 0.3 mL of mitochondria isolation buffer (0.35 M mannitol, 10 mM HEPES, pH 7.2, 0.1% bovine serum albumin) using a glass homogenizer. Unbroken cells and nuclei were pelleted by centrifugation (600 g for 5 min at 4°C). Then, the mitochondrial pellet was isolated by centrifugation (10,000 g for 10 min at 4°C) and the supernatant was collected and mixed with 25 μL of 10×SDS sample buffer. Samples (50 μL) were boiled and subjected to western blot analysis as previously described [13].

### Effect of inhibitors of p53 and necroptosis

When lung cancer cell lines A549 and H1299 reached 50–70% confluence, they were transfected with the B2 plasmid with PEI for 4 h. Then, the culture medium was changed and they were incubated for another 48 h. In inhibitor experiments, cells were treated with a P53 inhibitor (30 μM Pifithrin-α [52, 53], Sigma Aldrich, P4359) or a necroptosis inhibitor (40 μM necrostatin-1 [54], Sigma Aldrich, N9037).

### Mitochondrial staining assay

To track changes in mitochondrial morphology, cells were transfected as described above. After culture for 48 h, cells were stained with MitoTracker Red CM-H_2_XRos (Invitrogen) in accordance with the manufacturer’s instructions. Then, cells were analyzed by fluorescence microscopy, with excitation at 488 nm green fluorescence measured with a 515-nm long-pass filter, and with 510 nm excitation and red fluorescence measured with a 590-nm long-pass filter, as previously described [30].

### Intracellular ROS content of lung cancer cells

The generation of ROS was evaluated by a fluorescent-cytometry assay based on intracellular oxidation of H_2_DCFDA (Life Technologies, Carlsbad, CA, USA) [55]. Cells in the logarithmic growth phase were incubated in a 6-well plate overnight. Then, the medium was replaced with B2 transfection medium for 48 h. Cells were then washed with phosphate-buffered saline (PBS), resuspended at a concentration of 1 × 10^6^ cells/mL, and stained for 30 min at 37°C. Cells were observed by fluorescence microscopy, with excitation at 488 nm and measurement of green fluorescence using a 515-nm long-pass filter [30].

### Protein extraction and western blot analysis

After various times of incubation, cells were rinsed with 1×PBS, 3% BSA, and 0.1% Tween-20, then lysed with 0.05% SDS, boiled for 2 min, and centrifuged (10,000 g at 48°C for 10 min). The supernatant was diluted with 6×Laemmli loading buffer and boiled for 2 min prior to loading. Proteins were resolved by 10% sodium dodecyl sulfate–polyacrylamide gel electrophoresis, and electro-blotted onto nitrocellulose membranes. The membranes were incubated in a blocking solution (3% BSA, 0.1% Tween-20, 1×TBS) for at least 1 h at room temperature (RT). Immunoblotting was performed with the following antibodies overnight at 4°C: anti-FLAG primary monoclonal antibodies (Sigma), Bax, Bcl2, Bid, Bak, LC3, P53, P53 ser15, P53 ser392, P53 ser46, RIP3, beclin-1, and caspase-3 (Cell Signaling Technology). Then, membranes were washed with TBS and 0.1% Tween-20, and incubated for 1 h at RT with the secondary antibody (horse radish peroxidase, DakoCytomation) at a dilution of 1:2000. After washing, the membranes were developed using the enhanced chemiluminiscence system (ECL, Amersham Life Sciences). The signals were quantified using ImageJ software and ß-actin was used as a loading control [13, 30].

### Assays for apoptosis and necrosis

The Annexin V-FITC/Propidium iodide (PI) flow cytometric assay was used to measure early and late apoptosis, according to the manufacturer’s instructions (Annexin V-FITC/PI, Rocha). Briefly, A549 and H1299 cells were transfected with FLAG or FLAG-B2 plasmids for 48 h at 37°C, then washed twice with cold PBS, and centrifuged at 1000 rpm for 5 min. The harvested cells were resuspended in 200 μL binding buffer that contained 10 μL Annexin V-FITC. After 15 min, the cells were washed twice and resuspended in 300 μL binding buffer, and 10 μL of PI was added. Then, the cells were immediately analyzed by flow cytometry using a FACS Vantage cell sorter (Becton-Dickinson, San Jose, CA, USA). PI red fluorescence was measured using a 650-nm long-pass filter. Apoptotic and necroptotic cells have higher PI fluorescence (PI^+^) than intact cells (PI^−^). Each analysis examined at least 10,000 cells in the gated region, based on light scattering properties. Fluorescence data are displayed on one or two major scales, as previously described [13].

### 3D-structure prediction

SWISS-MODEL Repository (SMR) and Phyre2 web portal are a database of annotated 3D protein structure models generated by the automated SWISS-MODEL homology modeling pipeline [56, 57] and Phyre2 web portal system [58]. In the 3D-strcuture prediction, the full length RGNNV B2 (1-75 aa) and B2 mitochondrial targeting domain (36-61 aa) alone sequence for comparing from either SWISS-MODEL Repository system or Phyre2 web portal system. Two systems we have found that received very similar results. Then further confirmed the 3D-structure of protein B2 to published alpha-nodavirus protein B2 in dimer form structure [59] that still received the consistent result, have shown the alpha helix structure.

### Statistical analysis

All western blot images are representative of at least three independent experiments. The level of ROS production (H_2_DCFDA assay) and percentage of Annexin-V and PI-fluorescein–positive cells was determined by counting 200 cells per sample. Each result is expressed as the mean ± SEM. Data were analyzed using the paired or unpaired Student’s *t*-test, as appropriate. For comparison of group means, a *P* value less than 0.05 was considered statistically significant.
